# Key Mechanisms and Potential Targets of the NLRP3 Inflammasome in Neurodegenerative Diseases

**DOI:** 10.3389/fnint.2020.00037

**Published:** 2020-07-24

**Authors:** Yadi Guan, Fang Han

**Affiliations:** ^1^PTSD Laboratory, Department of Histology and Embryology, Basic Medical University, China Medical University, Shenyang, China; ^2^Department of Gastroenterology, Shengjing Hospital of China Medical University, Shenyang, China

**Keywords:** NLRP3 inflammasome, Alzheimer’s disease, Parkinson’s disease, amyotrophic lateral sclerosis, neurodegenerative disease

## Abstract

Neurodegenerative diseases are neuronal disorders characterized by the loss of a large number of neurons in the human brain. Innate immunity-mediated neuroinflammation actively contributes to the onset and progression of neurodegenerative diseases. Inflammasomes are involved in the progression of the innate immune response and are responsible for the maturation of caspase-1 and inflammatory cytokines during neuroinflammation. The nucleotide-binding oligomerization domain leucine-rich repeat and pyrin domain-containing protein 3 (NLRP3) inflammasome, which is one of the most intensively investigated inflammasomes, has been reported to play a key role in neurodegenerative diseases. Here, we reviewed the mechanisms, role, and latest developments regarding the NLRP3 inflammasome with respect to three neurodegenerative diseases: Alzheimer’s disease (AD), Parkinson’s disease (PD), and amyotrophic lateral sclerosis (ALS). Patient and animal model studies have found that abnormal protein aggregation of Aβ, synuclein, or copper–zinc superoxide dismutase-1 (SOD1), which are the main proteins expressed in the three diseases, respectively, can activate microglial cells, induce increased interleukin-1β (IL-1β) release, and activate the NLRP3 pathway, leading to neurodegeneration. In contrast, a deficiency of the components of the NLRP3 pathway may inhibit Aβ, synuclein, or SOD1-induced microglial activation. These studies indicate a positive correlation between NLRP3 levels and abnormal protein aggregation. However, in the case of ALS, not only microglia but also astrocytes express increased NLRP3 levels and contribute to activation of the NLRP3 pathway. In addition, in this review article, we also focus on the therapeutic implications of targeting novel inhibitors of the NLRP3 inflammasome or of novel drugs that mediate the NLRP3 pathway, which could play a role *via* NLRP3 in the treatment of neurodegenerative diseases.

## Introduction

Neurodegenerative diseases are neuronal disorders characterized by the loss of a large number of neurons in the human brain, for example, Alzheimer’s disease (AD), Parkinson’s disease (PD), amyotrophic lateral sclerosis (ALS), and Huntington’s disease. Although the location and etiology of these diseases differ, they share the common characteristic of neurodegeneration. AD and PD mainly occur in middle and old ages, so as the population ages, the incidences of AD and PD are increasing. It is generally believed that the prevalence of dementia in the population over 65 years old is 4% and that the annual incidence rate is 0.6% to 1.2%. The prevalence of PD is second only to that of AD, with PD mainly occurring in those of middle age or older at a prevalence of 2% in the population over 65 years old. However, Huntington’s disease and ALS can occur in patients of different ages, and there is no cure available for these diseases. One of the pathological hallmarks of neurodegenerative diseases is the aggregation of abnormal protein in the central nervous system (CNS). However, innate immunity-mediated neuroinflammation actively contributes to the onset and progression of neurodegenerative diseases (Labzin et al., 2018). Furthermore, inflammasomes have an important role to play in neuroinflammation and in neurodegenerative diseases (Heneka et al., 2013).

Inflammasomes are multiprotein complexes mainly located in the CNS, where they are found in immune cells, neural cells, microglia, and astrocytes (Freeman et al., 2017; Heneka et al., 2018; Song et al., 2018). They are integral parts of the innate immune response and are responsible for detecting and eliminating pathogen-associated molecular patterns (PAMPs), as well as danger-associated molecular patterns (DAMPs), subsequently secreting proinflammatory cytokines (Martinon et al., 2002). Various inflammasomes play critical roles in neurodegenerative diseases, especially the nucleotide-binding oligomerization domain leucine-rich repeat and pyrin domain-containing (NLRP) 3 inflammasome. The NLRP3 inflammasome can be activated by the abnormal protein aggregation that occurs in neurodegenerative diseases (Heneka et al., 2013; Wang et al., 2020). The resulting overexpression of proinflammatory cytokines can aggravate the chronic inflammatory response and pyroptosis in the CNS (Voet et al., 2019; Haque et al., 2020).

In this review article, we discuss the latest research developments regarding the NLRP3 inflammasome; its role in AD, PD, and ALS; and the therapeutic implications of targeting the NLRP3 inflammasome.

## Structures and Activation of the NLRP3 Inflammasome

The components of inflammasomes consist of sensors, the apoptosis-associated speck-like protein containing a caspase recruitment domain (ASC) protein, and procaspase-1. The sensors, which can detect PAMPs, DAMPs, and cytosolic double-stranded DNA, are classified into three types: nucleotide-binding domain and leucine-rich repeat-containing receptors (NLRs); absent in melanoma-like receptors (ALRs); and pyrin (Dubois et al., 2016). ASC links the pyrin domain (PYD) of the NLR, ALR, or pyrin to the caspase recruitment domain (CARD) of procaspase-1 (Lang et al., 2018; Voet et al., 2019). NLRP1, NLRP2, NLRP3, NLRP6, NLRP7, NLRP12, and NLR family CARD domain-containing 4 (NLRC4) all belong to the NLR family (Lamkanfi and Dixit, 2014; Lenart et al., 2016), while absent in melanoma 2 (AIM2) is a well-characterized ALR from a protein family containing four members in humans (Wang and Yin, 2017).

The NLRP3 inflammasome is one of the most intensively investigated inflammasomes and plays a vital role in innate immunity. It consists of an amino-terminal PYD, a central nucleotide-binding and oligomerization domain (NACHT), and a C-terminal leucine-rich repeat domain (LRR; Franchi et al., 2009). After the PYD of NLRP3 interacts with the PYD of ASC, the NLRP3 inflammasome is assembled and attracts caspase-1. The activation of the NLRP3 inflammasome requires a two-step process, comprising priming and then activation. Firstly, cells need to activate the nuclear factor-kappa B (NF-κB) pathway to upregulate the expression of NLRP3, caspase-1, and pro-interleukin-1β (pro-IL-1β), through the stimulation of toll-like receptors (TLRs; Toma et al., 2010; Qiao et al., 2012). Once primed, the NLRP3 complex is activated by multiple stimuli, including ionic flux, extracellular ATP, reactive oxygen species (ROS), and lysosomal rupture (Muñoz-Planillo et al., 2013; Minutoli et al., 2016; Li R. et al., 2018; Kelley et al., 2019).

There are two types of signaling pathways that activate the NLRP3 inflammasome: the canonical and noncanonical signaling pathways (Xiang et al., 2020). The canonical signaling pathway depends on caspase-1 and involves inflammasome complexes detecting pattern recognition receptor (PRR) proteins and inducing recruitment of procaspase-1. Procaspase-1 recruitment causes proximity-induced oligomerization and autoactivation, and release of active caspase-1 fragments (Voet et al., 2019). Consequently, caspase-1 cleaves biologically inactive pro-IL-1β and pro-IL-18 into the mature inflammatory cytokines IL-1β and IL-18, respectively (Akita et al., 1997; Rano et al., 1997). Caspase-1 also activates gasdermin D (GSDMD), which translocates to the plasma membrane and forms pores. Then IL-1β and IL-18 are transferred from the cytoplasm to the extracellular space through these pores, inducing a proinflammatory form of cell death known as pyroptosis, which exerts an inflammatory effect to cause further damage (Haque et al., 2020; Liang et al., 2020).

The noncanonical signal pathway is mainly dependent on mouse caspase-11 (of which caspase-4 and caspase-5 are the human counterparts). The noncanonical inflammasome is activated by lipopolysaccharide (LPS) in the cytosol released from gram-negative bacteria, such as *Escherichia coli*, *Citrobacter rodentium*, and *Vibrio cholera* (Hagar et al., 2013). The CARD motif of pro-caspase-11 (pro-caspase-4/-5) directly interacts with the lipid A tail of intracellular LPS (Yi, 2017), whereas the mature caspase-11 can induce pyroptosis and secretion of proinflammatory cytokines. Activation of caspase-11 in the noncanonical inflammasome can also induce activation of the canonical NLRP3 inflammasome, a process that is termed “noncanonical NLRP3 inflammasome activation” (Pellegrini et al., 2017).

## Role of NLRP3 in Alzheimer’s Disease

AD is mainly caused by the accumulation of amyloid-β (Aβ) plaques and neurofibrillary tangles (NFTs) in the brain, involving the increased production, oligomerization, and aggregation of Aβ peptides cleaved from the amyloid β-precursor protein (APP) by the β- and γ-secretase complexes. Aβ is the main component of extracellular senile plaques, which can cause neurotoxicity in AD (Hardy and Selkoe, 2002), triggering neurodegeneration and apoptosis, and leading to memory loss. In the past 15 years, numerous studies have focused on the role of the NLRP3 inflammasome in mediating neuroinflammation during the pathogenesis of AD. During this time spent studying the association between the NLRP3 inflammasome and AD, there have been two clear phases of research. Before 2013, most articles examined the role and regulatory pathway of NLRP3 in AD. From 2013 onwards, published articles have focused more on the therapeutic implication of targets and inhibitors of NLRP3 in AD.

Studies based on clinical data and animal experiments have both found that Aβ deposits can cause inflammasome activation in AD. Aβ activates microglial cells to produce IL-1β, which is a major outcome of NLRP3 inflammasome activation (Lamkanfi and Dixit, 2010; Lamkanfi, 2011). Levels of IL-1β level are significantly increased in the brain tissue, cerebrospinal fluid, and peripheral blood of AD patients (Tschopp and Schroder, 2010). Results from AD transgenic mice with Aβ treatment show that they also express high levels of caspase-1 and IL-1β in their brain tissue (Lue et al., 2001; Halle et al., 2008; Heneka et al., 2013). Microglia express higher levels of IL-1β especially around Aβ plaques (Apelt and Schliebs, 2001; Martinon et al., 2006). This overexpression of IL-1β can aggravate the chronic inflammatory response in the CNS (Meyer-Luehmann et al., 2008). Some additional elements such as particulate matter (PM) 2.5 or chronic cerebral hypoperfusion can accelerate the activation of the NLRP3 inflammasome and enhance inflammatory responses and neuronal damage in an AD model (Wang et al., 2018; Shang et al., 2019). Conversely, in an AD mouse model, inhibitors of NLRP3 or caspase-1 have been shown to significantly enhance the ability of microglia to clear Aβ deposits, reduce Aβ deposition, and improve cognitive impairment and hyperactive behavior (Heneka et al., 2013; Dempsey et al., 2017).

Furthermore, some studies have found that IL-1β can induce tau protein phosphorylation in the cortex of AD rats (Murakami et al., 2012). Thioredoxin-interacting protein (TXNIP) is an endogenous regulator of redox/glucose-induced stress and inflammation. Overexpression of TXNIP and its colocalization with IL-1β have been found near Aβ plaques and p-tau in the brains of AD patients (Li et al., 2019). The effects of inhibitors of caspase-1 or NLRP3 on Aβ deposits and behaviors in an AD model are reduced by decreasing the level of microglial pyroptosis. These findings suggest that NLRP3 and IL-1β play important roles in the pathophysiology of AD by regulating the pyroptosis, tau, or TXNIP pathways.

In general, in the AD brain, microglia phagocytose the increased amounts of Aβ and cause lysosomal damage. Disruption of lysosomes causes cytosolic release of cathepsin B, which is an endogenous signal for the NLRP3 inflammasome (Halle et al., 2008). NLRP3 activated by Aβ can induce ever-increasing production of IL-1β, promote “downstream” microglial synthesis, and release proinflammatory cytokines, and potentially neurotoxic factors, into the surrounding brain tissue (Heneka et al., 2013). These cytokines and cytotoxins further influence surrounding tissues and amplify the neurotoxic effects of Aβ.

Recently, studies have found that a number of drugs, such as donepezil, rivastigmine, and memantine, only partially improve cognitive and memory decline in patients with AD but do not fundamentally block or reverse the pathological changes resulting from the disease. Therefore, finding drugs that can reverse the development of AD is key to its treatment, with anti-inflammatory drugs potentially providing new possibilities for the treatment of AD. Although nonsteroidal anti-inflammatory drugs are effective in the treatment of AD model rats, their clinic application is limited owing to obvious side effects on the digestive system during the clinical treatment of AD (Wang et al., 2015). However, a large number of studies have clearly and uniformly concluded that the expression of Aβ deposits causes inflammasome activation, including activation of NLRPs and release of IL-1β in AD. Moreover, inhibition of NLRP3 can significantly inhibit amyloidosis and neuropathy and ameliorate cognitive behavior impairment in AD (Heneka et al., 2013; Dempsey et al., 2017). These studies reveal the possible roles of the NLRP3 inflammasome in the pathogenesis of AD and offer the possibility that an NLRP3 inhibitor could become a potential molecular target for improving AD-related symptoms and slowing AD progression at the neuroinflammatory level. A few NLRP3 inhibitors have already been produced, and their roles in AD have been investigated in disease models. MCC950 is a novel type of NLRP3 inhibitor that specifically inhibits the activation of NLRP3 in macrophages by inhibiting NLRP3-induced ASC oligomerization. *In vivo* experiments have shown that MCC950 significantly reduces the production of IL-1β/IL-18 and reduces the severity of disease in experimental autoimmune encephalomyelitis mice. MCC950 reduces the accumulation of Aβ in brain tissue and reduces the behavioral abnormalities of amyloid precursor protein (APP)/presenilin-1 (PS-1) transgenic mice (Dempsey et al., 2017; Mao et al., 2017). JC-124, a rationally designed NLRP3 inflammasome inhibitor, decreases levels of Aβ deposition and of soluble and insoluble Aβ1–42 in the brains of CRND8 transgenic mice with AD-related deficits. The purinergic 2 × 7 (P2 × 7) receptor antagonist, which is an inhibitor of the P2 × 7/NLRP3/caspase-1 pathway in microglia, might exert an anti-neuroinflammatory effect and be applicable for treating early-stage AD (Thawkar and Kaur, 2019).

Besides NLRP3 inhibitors, a number of novel anti-inflammatory and anti-allergy drugs might contribute to the treatment of AD *via* the NLRP3 pathway. OLT1177 is a novel drug for treating arthritis, which reduces neutrophil infiltration into the peripheral blood, reduces joint swelling, and inhibits IL-1 secretion in animal models of arthritis (Marchetti et al., 2018b). *In vitro* experiments suggest that OLT1177 inhibits the activation of NLRP3-mediated inflammation. Results from *in vivo* tests suggest that OLT1177 reduces the activity of caspase-1 and inhibits the production of IL-1 by direct binding to NLRP3 and inhibiting ATPase activity (Marchetti et al., 2018a,b). Meanwhile, tranilast is an anti-allergy drug that also has an anti-inflammatory effect and inhibits IgE-induced histamine secretion from mast cells (Darakhshan and Pour, 2015). Tranilast is a specific NLRP3 inhibitor that directly binds to the intermediate domain of NLRP3 and inhibits the oligomerization of ASC (Huang et al., 2018). *In vivo* experiments have also corroborated the preventive and therapeutic effects of tranilast on NLRP3 inflammation-related diseases (Huang et al., 2018).

Recently, some studies have shown that Chinese traditional medicines have advantages for the treatment of AD because of their multitarget pharmacological activities. Among these medicines for AD treatment, a number play immunomodulative or anti-inflammatory roles in treating AD, including icariin, rhodiola, poria cocos, total glycosides of Radix Paeoniae Alba, atractylodes, ginseng, and yuanzhi. Recent studies have found that the effects of these medicine are mediated through NLRP3; for example, the protective effect of *Epimedii folium* and Curculiginis Rhizoma on AD has been regulated by the inhibitions of the NF-κB/mitogen-activated protein kinase (MAPK) pathway and the NLRP3 inflammasome (Lan et al., 2017). Meanwhile, high-dose modified buwang-san ameliorates learning and memory deficits and attenuates neuroinflammation in the hippocampus of an AD mouse model by inhibiting the expression of NLRP3, caspase-1, and IL-1 (HE Ling-Ling et al., 2020). In addition, electroacupuncture, on the basis of the theory of traditional Chinese medicine, may improve spatial learning/memory and inhibit the inflammatory reaction in an animal model of AD by reducing the expression of IL-1β and NLRP3 inflammasome-related proteins (Jiang et al., 2018).

## Role of NLRP3 in Parkinson’s Disease

PD is an age-related neurodegenerative disorder characterized by progressive degeneration of dopaminergic (DA) neurons in the substantia nigra (SN) and accumulation of Lewy bodies, which are constituted of fibrillar α-synuclein (α-syn; Codolo et al., 2013; Petrucci et al., 2014). Numerous recent studies have indicated that inflammasomes play an important role in the progression of PD.

Research indicates that NLRP3, ASC, caspase-1, and IL-1β are increased in the peripheral blood mononuclear cells and plasma of PD patients compared with those of age-matched healthy controls (Zhou et al., 2016; Chatterjee et al., 2020; Fan et al., 2020). Levels of IL-1β in plasma have a positive correlation with the Hoehn and Yahr staging scale and Unified Parkinson’s disease Rating Scale (UPDRS) part III scores (Fan et al., 2020). Both IL-1β and IL-18 levels were found to be higher in the cerebrospinal fluid of PD patients than in that of control subjects (Zhang et al., 2016). Concentrations of IL-1β in the striatal regions, of cleaved caspase-1 and ASC in the SN, and of caspase-1 in mesencephalon were significantly higher in parkinsonian patients compared with normal group (Mogi et al., 1994; Gordon et al., 2018; von Herrmann et al., 2018). High mRNA and protein expression levels of NLRP3 inflammasome components were observed in LPS-, 6-hydroxydopamine- (6-OHDA), MPTP-, and MPTP/p-induced PD rats (Mao et al., 2017; Qiao et al., 2018). NLRP3 deficiency has been shown to alleviate motor dysfunction and loss of DA neurons in MPTP-treated mice (Lee et al., 2019). MPTP/p treatment elevates expression of caspase-1 in the mouse midbrain, whereas caspase-1 knockout ameliorates DA neuronal loss and dyskinesia induced by MPTP/p (Qiao et al., 2017). Inhibiting the downstream pathway of the NLRP3/caspase-1/IL-1β axis using Ac-YVAD-CMK, a caspase-1 inhibitor, improves the number of DA neurons in the SN and alleviates symptoms in both LPS- and 6-OHDA-induced PD rats (Mao et al., 2017). Furthermore, administration of IL-1 receptor antagonist (IL-1Ra) attenuates MPTP-induced PD phenotypes in mice (Lee et al., 2019). Therefore, these studies show that NLRP3 inflammasome activation is involved in the pathogenesis of PD.

The histopathological hallmark of PD is Lewy bodies, which are mainly composed of fibrillar aggregates of α-syn, is a presynaptic protein, which plays a crucial role in the pathogenesis of PD. Both oligomers and phosphorylated α-syn in the peripheral blood of PD patients have been shown to be significantly elevated (Fan et al., 2020). A number of studies have revealed a strong association between α-syn and the NLRP3 pathway. Plasma α-syn levels show a positive correlation with both UPDRS part III scores, and plasma NLRP3 and IL-1β levels (Chatterjee et al., 2020; Fan et al., 2020). Briefly, fibrillar α-syn induces NLRP3-caspase-1 complex activation followed by secretion of IL-1β in peripheral blood mononuclear cells, monocytes, microglia, and astrocytes (Chatterjee et al., 2020; Wang et al., 2020), with this proinflammatory cytokine further influencing brain tissue and increasing the inflammatory response. Caspase-1 then causes the truncation and aggregation of α-syn, and α-syn enters microglial cells in an endocytosis-dependent manner and subsequently activates two important innate receptors in the TLR2 (TLR4)/NF-κB and NLRP3/caspase-1 pathways (Gustot et al., 2015; Fan et al., 2016; Zhou et al., 2016). The truncation-induced aggregation of α-syn has been shown to be toxic to neuronal cultures (Wang et al., 2016). Caspase-1 deficiency significantly inhibits α-syn-induced microglial activation and IL-1β production (Zhou et al., 2016).

Other elements and factors also affect the activation of α-syn by the inflammasome: (1) fyn, a nonreceptor Src family tyrosine kinase, contributes to α-syn-induced NLRP3 inflammasome priming *via* PKCδ-mediated NF-κB activation (Panicker et al., 2019); (2) autophagy also participates in α-syn-induced neuroinflammation, with α-syn significantly increasing the autophagy-associated molecule Atg5, and the autophagy inhibitor 3-MA inhibiting α-syn-mediated activation of NLRP3/caspase-1/IL-1β (Wang et al., 2020); and (3) microRNAs, which have been recently recognized as crucial regulators of inflammasomes in PD models. MicroRNA-30e (miR-30e) ameliorates neuroinflammation in the MPTP model of PD by directly targeting NLRP3 and inhibiting the activation of the NLRP3 inflammasome (Li D. et al., 2018). MicroRNA-135b (miR-135b) plays a protective role in the MPP+-induced PD model *in vitro* *via* the inhibition of forkhead Box 1 (FOXO1)/NLRP3/caspase-1-mediated pyroptosis (Zeng et al., 2019). NLRP3 is a target gene of microRNA-7 (miR-7), with injection of miR-7 mimics into mouse striatum notably suppressing NLRP3 inflammasome activation and attenuating DA neuron degeneration in MPTP-induced PD model mice (Zhou et al., 2016). These microRNAs might therefore be effective therapeutic targets for PD.

A number of exogenous compounds have been shown to alleviative NLRP3 inflammasome-mediated neuroinflammation in PD models. Peroxisome proliferator-activated receptor β/δ (PPARβ/δ) agonists have been shown to suppress the inflammatory reaction and NLRP3 inflammasome activation. Administration of GW501516, a selective PPARβ/δ agonist, *via* intracerebroventricular infusion reduces movement impairment and attenuates DA neurodegeneration in the midbrain of PD mice (Chen et al., 2019). The potent sphingosine-1-phosphate receptor antagonist fingolimod (FTY720) significantly attenuates MPTP-induced PD progression and reduces the loss of DA neurons by inhibiting NLRP3 inflammasome activation (Yao et al., 2019). Traditional Chinese medicines have been proved to possess anti-inflammatory properties in relation to PD. Tenuigenin (a component of *Polygala tenuifolia*), Astragaloside IV (a component of *Astragalus membranaceus*), and bushen-yizhi formula (composed of common Cnidium fruit, tree peony bark, ginseng root, Radix Polygoni Multiflori preparata, Barbary wolfberry fruit, and Fructus Ligustri Lucidi) were shown to significantly ameliorate DA neuron degeneration and alleviate motor impairment by suppressing NLRP3 inflammasome activation in the MPTP mouse model (Fan et al., 2017; Mo et al., 2018; Leng et al., 2019). Based on these findings, medicines and exogenous compounds that target the NLRP3 inflammasome may offer novel therapeutic directions for treating PD.

## Role of NLRP3 in Amyotrophic Lateral Sclerosis

ALS is a progressive neurodegenerative disease caused by the deterioration of motor neurons in the brain and spinal cord. Among the cases of ALS, more than 90% are sporadic ALS (sALS) patients, and 5–10% are familial ALS (fALS) patients. Most fALS cases involve autosomal genetic diseases, such as superoxide dismutase-1 (SOD1) and nerve microfilament defects. Neuroinflammation is believed to significantly contribute to ALS disease progression (Byrne et al., 2011; Hardiman et al., 2011; Morgan and Orrell, 2016). Inflammation-induced neurotoxicity causes the activation of glial cells, including microglia and astrocytes (Philips and Robberecht, 2011), the activation of which leads to IL-1β production, causing further motor neuron death (Meissner et al., 2010). Inflammasome activation and upregulation of NLRP3 and its inflammasome components, caspase-1 and IL-1β, have been observed in ALS patients and in mouse models of ALS (Bellezza et al., 2018), suggesting that the NLRP3 complex plays key roles in ALS pathology (Meissner et al., 2010).

The SOD1G93A mutation was used to create the SOD1G93A mouse (Ince et al., 2011), which is the most widely used model of fALS. This mutation decreases the folding stability of SOD1, inducing the formation of protein aggregates (Philips and Robberecht, 2011) that play an important role in SOD1-mediated pathogenesis of ALS (Alexianu et al., 2001). High levels of caspase-1 and IL-1β in microglia contribute to disease progression in the mouse SOD1G93A model, suggesting a role for microglial NLRP3 in ALS (Alexianu et al., 2001; Philips and Robberecht, 2011). LPS activates caspase-1, leading to an increase in IL-1β release in SOD1G93A mice (Meissner et al., 2010). LPS also increases levels of ROS and nitric oxide, which forms peroxynitrite, leading to protein nitration, and concomitantly increasing protein nitrify in the spinal cord of pre-symptomatic SOD1G93A mice (Sargsyan et al., 2011; Bellezza et al., 2018). The reduction in peroxynitrite levels results in a decrease in caspase-1 activity. These studies indicate that peroxynitrite formation induced by oxidative/nitrosative stress may play a critical role in inflammasome activation and might be exploited as potential therapeutic target for ALS (Zhao et al., 2010; Jo et al., 2016).

However, other results from ALS patients and from SOD1G93A mice indicate that microglia do not express NLRP3. Caspase-1 activation and IL-1β production in microglia have been shown to occur independently of NLRP3 (Meissner et al., 2010). However, microglial NLRP3 upregulation has been observed in another ALS model, the tar DNA-binding protein 43 (TDP-43) mouse, suggesting that the TDP-43 mutant might activate microglial inflammasomes in an NLRP3-dependent manner (Ince et al., 2011). TDP-43 is a major hallmark of the protein aggregates induced by SOD1 in ALS patients (Neumann et al., 2006) and translocates from the nucleus to the cytoplasm as part of the pathogenesis of the disease (Barmada et al., 2010). Mutations in TDP-43 (e.g., TDP-43Q331K) also lead to fALS (Sreedharan et al., 2008). Transgenic TDP-43Q331K mice have increased microglial activation and motor neuron degeneration (Lee et al., 2018). These studies indicate that pathological ALS proteins activate the microglial NLRP3 inflammasome.

In addition, besides microglia, astrocytes are also capable of contributing to innate immune signaling by increasing their release of cytokines, including IL-1β and IL-18 (Dong and Benveniste, 2001; Kipp et al., 2007; van Neerven et al., 2010). Gene expression profiling of astrocytes suggests that inflammatory mechanisms are activated early in ALS. Spinal cord astrocytes have been identified as the major cell type expressing NLRP3 components. In human ALS tissue, increased levels of NLRP3, ASC, IL-18, and active caspase-1 have been found in astrocytes (Haidet-Phillips et al., 2011; Johann et al., 2015).

In general, these findings suggest that astroglial NLRP3 inflammasome complexes are critically involved in neuroinflammation in ALS. In ALS, microglia and astrocytes switch from a neuroprotective to a proinflammatory phenotype by releasing potentially neurotoxic cytokines.

## Conclusion and Future Research Direction

A number of studies have been focused on understanding the role of the inflammasome in neurodegenerative diseases. Increasing evidence indicates a link between NLRP3 and neurodegenerative diseases, although the exact roles and mechanisms by which the NLRP3 inflammasome regulates neurodegenerative diseases remain unclear and require further confirmation. Most importantly, inhibition of NLRP3 in animal models reduces the inflammatory response, decreases abnormal protein deposition, and corrects behavioral abnormalities associated with neurodegenerative diseases, suggesting the possibility of targeting NLRP3 inflammasome to treat such diseases. However, many important topics still need to be explored, such as how the activation or inhibition of the NLRP3 inflammasome influences neurodegenerative diseases. The solution of such questions is important for studying the pathogenesis of neurodegenerative diseases and for providing more evidence and targets for exploring novel treatments. Despite the fact that inhibition of NLRP3 inflammasome activation has been shown exert beneficial effects in animal models of neurodegenerative diseases, the effectiveness and safety of specific NLRP3 inflammasome inhibitors in patients have yet to be verified in clinical trials.

## Author Contributions

FH and YG contributed to the conception and organization and wrote the first draft of the manuscript. FH contributed to manuscript revision. All authors approved the submitted version.

## Conflict of Interest

The authors declare that the research was conducted in the absence of any commercial or financial relationships that could be construed as a potential conflict of interest.

## Abbreviations

NLRP3: nucleotide-binding oligomerization domain leucine-rich repeat and pyrin domain-containing protein 3
AD: Alzheimer’s disease
PD: Parkinson’s disease
ALS: amyotrophic lateral sclerosis
α-syn: α-synuclein
SOD1: superoxide dismutase-1
IL-1β: interleukin-1β
CNS: central nervous system
PAMPs: pathogen-associated molecular patterns
DAMPs: danger-associated molecular patterns
ASC: apoptosis-associated speck-like protein containing a caspase recruitment domain
NLRs: nucleotide-binding domain and leucine-rich repeat containing receptors
ALRs: absent in melanoma-like receptors
PYD: pyrin domain
CARD: caspase recruitment domain
AIM2: absent in melanoma 2
NACHT: nucleotide-binding and oligomerization domain
LRR: leucine-rich repeat domain
TLRs: toll-like receptors
PRR: pattern recognition receptor
GSDMD: gasdermin D
LPS: lipopolysaccharide
Aβ: amyloid-β
NFTs: neurofibrillary tangles
TXNIP: thioredoxin-interacting protein
SN: substantia nigra
UPDRS: Unified Parkinson’s Disease Rating Scale
MPTP: 1-methyl-4-phenyl-1,2,3,6-tetrahydropyridine
6-OHDA: 6-hydroxydopamine
PPARβ/δ: peroxisome proliferator-activated receptor β/δ
ROS: reactive oxygen species.
